# Tooth loss in Sjögren’s syndrome patients compared to age and gender matched controls 

**DOI:** 10.4317/medoral.22545

**Published:** 2018-09-28

**Authors:** Floor Maarse, Derk H. Jan Jager, Tim Forouzanfar, Jan Wolff, Henk S. Brand

**Affiliations:** 1 Department of Maxillofacial Surgery and Oral Pathology, VU University Medical Center, Amsterdam Movement Sciences, The Netherlands; 2 Center for Special Care Dentistry (Stichting Bijzondere Tandheelkunde), Amsterdam, The Netherlands; 3 Department of Oral Biochemistry, Academic Center for Dentistry Amsterdam (ACTA), Amsterdam, The Netherlands; 4 Department of Oral and Maxillofacial Surgery and Oral Pathology/3D Innovation Lab, VU University Medical Center, Amsterdam Movement Sciences, The Netherlands; 5 Department of Oral Health Sciences, KU Leuven & University Hospitals Leuven, Leuven, Belgium

## Abstract

**Background:**

To analyze the prevalence and location of tooth loss in Sjögren’s syndrome (SS) patients and compare them with an age- and gender-matched control group.

**Material and Methods:**

Dental charts and x-rays of 108 (SS) patients were retrieved from an academic dental center and special care dentistry department. For each SS patient, an age- and gender-matched non-SS patient was randomly selected. Medication, number of extractions and date and location of extractions were assessed. Differences between SS and non-SS patients were analyzed using Mann-Whitney U tests, Chi-square tests and Fisher’s exact tests.

**Results:**

Significantly more SS patients were edentulous compared to the non-SS group (14.8% versus 1.9%, *p* = 0.001). SS patients had a 61% higher risk to have experienced one or more extractions than control patients. In the SS group, there was a non-significant tendency for more maxillary teeth to have been extracted than mandibular teeth (42:34). In the control group, the number of extractions in the maxilla and mandible were comparable (21:20). When divided into sextants, the number of SS patients with one or more extractions was significantly higher than for non-SS patients for each sextant (*p* = 0.001 to *p* = 0.032). The largest difference in the proportion of patients with one or more extractions between the SS and non-SS patients occurred in the upper anterior sextant (3.4 times more frequent).

**Conclusions:**

SS patients are more prone to experience dental extractions compared to patients without SS. It could be speculated that this is related to a decreased salivary secretion.

** Key words:**Sjögren’s syndrome, dentition, edentulism, extractions, dental health.

## Introduction

Sjögren’s syndrome (SS) is a progressive autoimmune disorder that causes chronic inflammation and irreversible damage to the exocrine glands (1). The syndrome is classified as either primary or secondary SS (pSS, sSS). Secondary SS is accompanied by other autoimmune diseases such as rheumatoid arthritis, systemic sclerosis, antiphospholipid syndrome or systemic lupus erythematosus (2). SS is more prominent in women than in men (9:1 ratio) (3).

The parotid, submandibular and the sublingual glands induce the most salivary flow in the oral cavity. In addition, approximately 800 accessory glands contribute to the saliva flow. In SS patients, the salivary flow is impaired due to glandular destruction. The current theory is that a combination of sex hormones, genetic disposition and environmental factors are involved in the pathogenesis of SS (4). The initial glandular inflammation is triggered by an immune response that subsequently activates T and B cells. This immune response leads to an autoantibody activation that causes tissue destruction and thereby reduces the salivary flow. However, it must be noted that the full pathogeneses of SS still remain to be discovered and nowadays no scientifically proven therapy exists for SS (5,6).

In SS patients, the sublingual and submandibular glands are often the first to be affected and in most cases de-gradation of the salivary glands occurs bilaterally (7-9). Due to its progressive nature, SS causes a gradual reduction in the salivary flow. When the unstimulated saliva secretion rate falls below 0.1 mL/min, this is usually regarded as hyposalivation (10). Hyposalivation can cause eating and swallowing problems, lack of taste, speech problems, and bacterial or fungal inflammations of the oral mucosa (11,12). Furthermore, hyposalivation can result in reduced retentiveness of prosthetic appliances and the dry oral mucosa can be very sensitive, which makes it difficult or even impossible for SS patients to wear mucosal supported removable prostheses.

Saliva is crucial for the maintenance of the oral health as it contains a variety of immunoglobulins and antibacterial proteins that protect and lubricate the mouth and throat (13). Furthermore, saliva acts as a buffer and supports the re-mineralization of enamel (14).

Due to the lower salivary pH, reduced buffer capacity and decreased anti-bacterial potency of saliva, patients with SS have a higher risk of developing dental caries and erosion, in particular the cervical, buccal and incisal sides of the teeth are commonly affected (13). Furthermore, recent studies have reported that SS patients might have a higher risk of developing periodontal disease. However current literature on the topic remains contradictory (15-18).

To date there is still little information on the average tooth loss in SS patients (19). Such information would be beneficial because it would offer clinicians the possibility to predict the location of tooth loss and adjust their treatment plans accordingly. Furthermore, such information could contribute to the development of dental protocols for SS patients. Based on clinical experience it can be hypothesized that SS patients lose their teeth faster than non-SS patients as a consequence of the decreased salivary secretion. Therefore, the aim of this study was to evaluate tooth loss in SS patients and compare this to a non-SS age- and gender-matched control group.

## Material and Methods

- Study design

An, age- and gender-matched, retrospective cohort was used in this study. The study population comprised 108 SS patients and a control group of 107 age and gender matched non-SS patients. All SS patients included in the study were diagnosed with primary or secondary SS according to the American-European Consensus Group classification Criteria for SS (20). The minimum follow-up period was 3 years. All patients with a history of radiation therapy in the head and neck region, HIV, sarcoidosis, lymphoma or graft-versus-host disease, were excluded. This study followed the Declaration of Helsinki on medical protocol and ethics and the data was collected in accordance to the guidelines of the Medical Ethical Committee of the VU University Medical Center. The Ethics Review Committee of the VU University Amsterdam confirmed that the Medical Research Involving Human Subjects Act (WMO) does not apply to this study. The reporting of this study conforms to the STROBE statement (21).

Variables The primary outcome measure was the number and location of tooth extractions. Patient age, gender, medication, and date of extractions were assessed.

Data collection methods Case report forms (CRFs) were designed to collect data in a standardized manner. Three data abstractor, with specialized knowledge in the field of SS and the research question (FM,JV,AW), abstracted data from all patients records. In order to prevent incorrect transfer of patient data from the medical records to the case report forms, random checks were performed prior to data entry. This was done according to the 100-20 rule in which 100% of the data is checked in 20% of the CRF’s and 20% of the most important data was checked in 100% of the CRF’s to prevent mistakes in data retrieval (21,22).

- Data analysis As this was not a prospective but a retrospective case-series study, in which a convenience sample comprising all patients from a dental school was described, a sample size analysis was not performed. Differences between SS and control patients were analyzed using Mann-Whitney U-test, Chi-square tests and Fisher’s exact tests. A significance level (α) of 0.05 was chosen. Statistical analysis was performed using SPSS, version 23.0 (IBM Corp, Armonk, NY, USA).

## Results

- Patients

The size of the study population was based on the number of patients with SS who fulfilled the aforementioned inclusion criteria. Most of the patients (89%) were female. The average age of the patients was 66.5 years (range 34.7-94.2). The study population consisted of 57 patients diagnosed with pSS (52.8%) and 51 patients diagnosed with sSS (47.2%). When comparing the age of the pSS with the sSS patients, the latter group was significantly older (*p*=0.005).

The SS group comprised of patients who had been enrolled at the Academic Center for Dentistry Amsterdam (ACTA) or the Center for Special Care Dentistry Amsterdam (Stichting Bijzondere Tandheelkunde), for an average of 8.6 years (range 1.4–22.0 years). The control group consisted of patients with an average age of 65.0 years (range 30.6 – 92.9), that had been enrolled at ACTA for an average of 13.0 years, (range from 2.3-25.3 years). In the control group, also 89% of the subjects were female.

- Extractions

**Figure 1:**
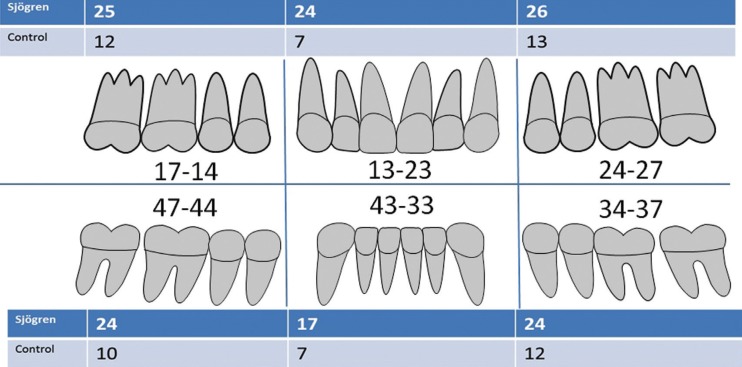
Number and location of extraction in the Sjögren’s syndrome patients and control subjects.

Patients with SS had a 61% higher risk of one or more extractions than patients in the control group ( Table 1, Fig. 1). The mean number of extractions were significantly higher in the SS group (3.18 ± 6.33 versus 0.77 ± 1.77) versus the control group. These results were found both in the maxilla (1.75 ± 3.54 versus 0.38 ± 1.04) and the mandible (1.43 ± 3.11 versus 0.38 ± 1.04) ( Table 2). Additionally, the number of extractions per sextant was significantly higher in the SS group when compared with the control group ( Table 2). In the sextant 13-23 the proportion of patients with at least one extraction (≥ 1) in the SS group was 3.4 times higher than in the control group, compared with 1.7-2.4 in the other sextants. When comparing the pSS with the sSS patients, the sSS patients had significantly more often extractions in the anterior maxilla sextant 13-23 (*p*=0.03; chi2 test) ( Table 3).

**Table 1:**
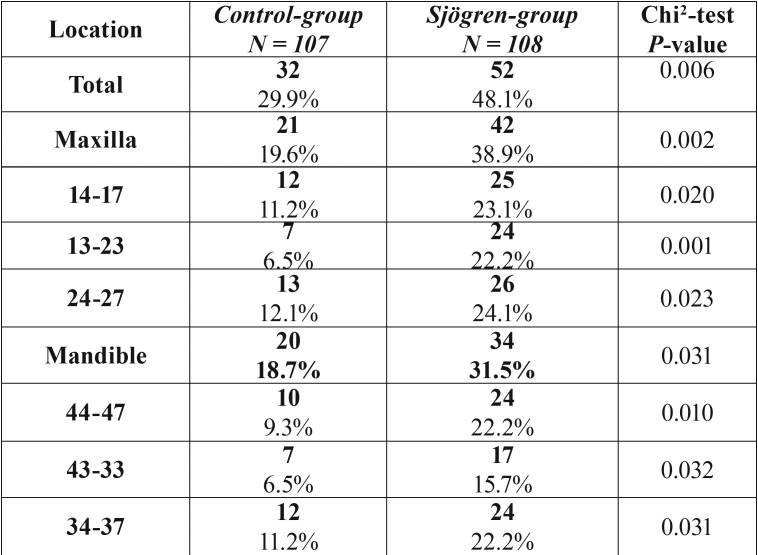
Number of Sjögren’s syndrome patients and control subjects with ≥1 extraction (s) distributed according to the location.

**Table 2:**
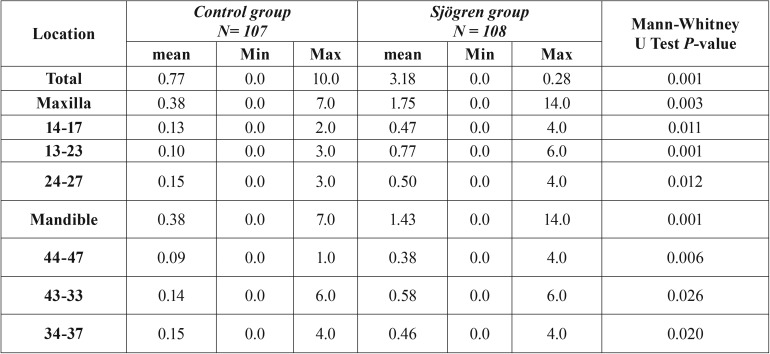
Average number of extractions of Sjögren’s syndrome patients and control subjects, distributed according to the location.

**Table 3:**
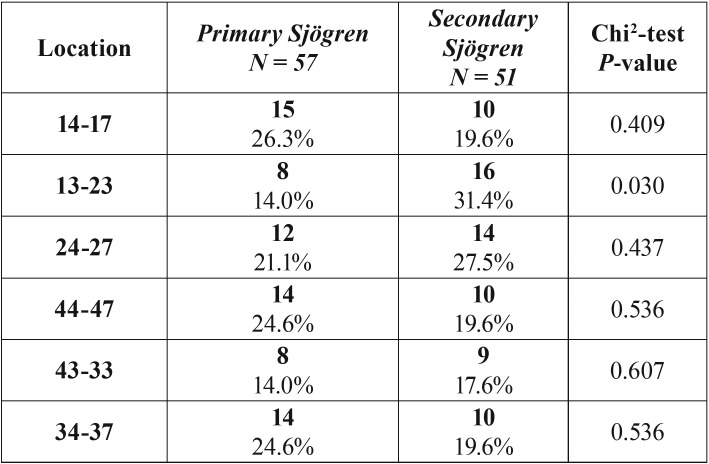
Number of patients with primary (pSS) and secondary Sjögren’s syndrome (sSS) with ≥1 extraction (s) distributed according to the location.

In the SS group, there was a non-significant tendency for more maxillary teeth to have been extracted than mandibular teeth (42:34). In the control group, the number of extractions in the maxilla and mandible were comparable (21:20).

Significantly more patients in the SS group (pSS + sSS) (14.8%) were edentulous compared to the control group (1.9%), (*p* = 0.001).

- Medication 

In total 43.5% (n=47) of the patients in the SS group suffered from systemic disorders such as rheumatic diseases. In the control group hypertension was the most common systemic disorder (20.0%), followed by cardiovascular disease (13.1%) and hypercholesterolemia (12.1%). Medication was prescribed more frequently in the SS group than in the control group ( Table 4). SS patients frequently used prescription medication for cardiovascular, anti-inflammatory, gastrointestinal disorders and antidepressants. Patients diagnosed with SS more frequently used anti-inflammatory drugs (*p* < 0.001); antidepressants (*p* < 0.001); diuretics (*p* = 0.009), thyroid medication (*p* = 0.03) or drugs that can induce a dry mouth than non-SS patients ( Table 4).

**Table 4:**
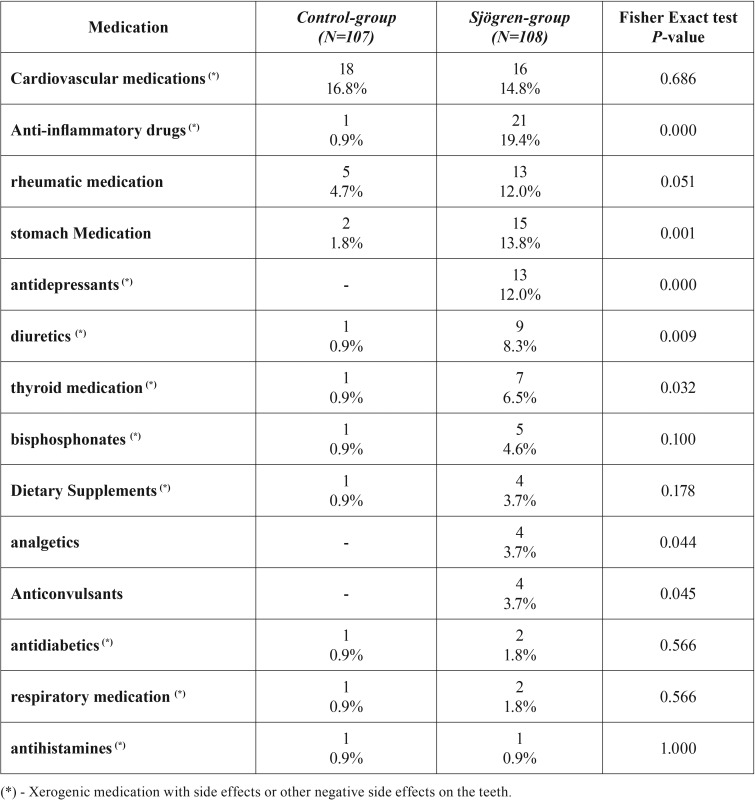
Medication use of Sjögren’s syndrome patients and control subjects.

## Discussion

This study demonstrated that SS patients are more prone to tooth loss than individuals not diagnosed with Sjögren’s syndrome. In addition, the results of this study shows that extractions are more prone in some area’s then in others with the highest number of extractions in the anterior maxilla. These results are in good agreement with a previous study on the prevalence of dental caries amongst SS patients (19). The same study reported that 63% of all study patients with SS were edentulous before the age of 45, compared to 10% in the control group. Another study reported that the number of missing or restored teeth was significantly higher in patients with SS than in a control group (23). The SS patients in our study were also more prone to lose their teeth over a shorter period of time than the control group.

The earlier tooth loss observed in the SS group could be related to reduced salivary flow. In this context, a previous study on the effects of radiotherapy in the head and neck region demonstrated that reduced salivary flow commonly results in an increased susceptibility to caries (24). Saliva promotes the remineralization of teeth and salivary proteins protect the enamel surface against erosion and caries. Furthermore, saliva has a buffering function, and contains enzymes and peptides with antibacterial effects. In patients with SS, the salivary flow is not only reduced but the protein concentration and glycosylation are also altered (25,26). This subsequently influences the protective characteristics of saliva, and its ability to protect and maintain oral health (27). Multiple studies have reported that mucins are especially vital for binding lubricants to oral surfaces (25,28,29). This not only prevents a sense of dry mouth (xerostomia), but also inhibits bacteria from binding to oral surfaces. Moreover, patients suffering from SS have a reduced mucin concentration or functionally impaired mucins, which makes it easier for bacteria to attach to the oral surfaces and cause caries and infections. This might provide an explanation for the increased dental caries and early tooth loss in SS patients (25,28,29). Unfortunately, in this study salivary flow and composition were not available.

The enhanced tooth loss observed in the SS patients could have resulted from periodontal problems. Previous studies reported conflicting results concerning the risk of periodontal disease in SS patients. A study focusing on plaque, bleeding and calculus indexes, as well as probing depth and attachment levels, reported no statistical differences between SS patients and control patients (18). A similar study reported that SS patients had higher plaque index scores, higher decayed/missing/filled surface scores, increased alveolar bone loss, deeper clinical attachment levels, and increased pockets. It was concluded that the risk of acquiring adult periodontitis was 2.2 times higher in SS patients than controls (17). A more recent study that focused on the prospective evaluation of dental and periodontal status in patients with suspected SS reported that SS patients had a significantly higher plaque index and bleeding on probing. However, there was no significant difference in the probing depths between SS patients and control patients (16). Taken together, this indicates that there is still no conclusive evidence that SS enhances periodontal disease.

In addition to SS, certain classes of drugs can induce hypo-salivation by targeting neurotransmitters and receptors. Drugs that inhibit neurotransmitter binding to acinar membrane receptors or that interfere with ion transport pathways may negatively affect the quantity as well as the quality of saliva (30). Many of the drugs used by the SS patients and the control subjects in the present study may have had such an effect ( Table 4). In the SS group, significantly more xerogenic drugs were used than in the control group. This might have further reduced the salivary flow rate in the SS patient group.

Significant more teeth were lost in the anterior maxilla of the sSS group compared to the anterior maxilla of the pSS group. This could be explained by the fact that the patients in the sSS group where significantly older than the pSS group. These results are similar to a study that reported tooth loss and edentulism increase with age (31). A second explanation could be that sSS is often associated with rheumatoid arthritis, which may negatively affect the oral health of these patients. In this context, a study reported that patients with rheumatoid arthritis have impaired mobility of the shoulders and wrists, which may subsequently affect oral care (32). Both age and impaired oral health could potentially contribute to the premature loss of teeth in patients with secondary SS.

Noticeable is the similarity of the observed difference of the enhanced tooth loss in the anterior maxilla when both the SS groups are compared to the control patients. Several studies have found not only the lowest film velocities and biofilm in this region but also the lowest stimulated amylase in the anterior regions (33-35). It could be hypothesized that if the anterior region of the maxilla is already the least lubricated region in normal patients, that this area is also the first to be affected when salivary flow decreases.

A potential limitation of the present study is that the included patients were treated in specialized dental centers to which patients are referred to for the treatment of more severe and/or oral problems. As a result, it could be hypothesized that the average number of extractions in our SS group is higher than the number of extractions in the average Sjögren patient. Another limitation is that most patient records lacked information on whether preventive oral care was provided, disease duration and salivary flow rates. This useful information was unfortunately not available, and could not be measured due to the retrospective nature of our study.

## Conclusion

In conclusion, the present study demonstrated that extractions are more frequent in SS patients compared with non-SS patients. In addition, the results of this study shows that extractions are more prone in some area’s then in others with the highest number of extractions in the anterior maxilla. Data collected in this study could contribute to the development of protocols for SS patients regarding oral hygiene as well as prosthodontic and implant treatments.
